# Comparison of bilateral ligaments after unilateral anterior cruciate ligament reconstruction: Based on magnetic resonance imaging analysis

**DOI:** 10.1371/journal.pone.0312704

**Published:** 2024-10-24

**Authors:** Zirong Huang, Jiamin Liang, Hongliang Gao, Kan Chen, Mingjin Zhong, Weimin Zhu

**Affiliations:** 1 Department of Sports Medicine, Shenzhen Second People’s Hospital, The First Affiliated Hospital of Shenzhen University, Shenzhen, Guangdong, China; 2 Guangdong Key Laboratory of Tissue Engineering, Shenzhen Second People’s Hospital, The First Affiliated Hospital of Shenzhen University, Shenzhen, Guangdong, China; 3 Department of Spinal Surgery, The 940 Hospital of Joint Service Support Force of Chinese PLA, Lanzhou, China; Casa di Cura Villa Erbosa, ITALY

## Abstract

**Objective:**

This study quantitatively assessed postoperative changes in graft inclination angle and femorotibial position after ACL reconstruction using MRI, to identify reliable indicators for evaluating knee stability.

**Methods:**

A retrospective analysis of 50 cases of ACL reconstruction from June 2019 to June 2020 included clinical outcome measures. MRI assessed graft/ACL inclination angles, medial/lateral anterior tibial translation (ATT), and femoral/tibial rotation angles on both surgical and contralateral sides. Femorotibial angle (FTA) and rotational tibial subluxation (RTS) were calculated for comparing MRI results.

**Results:**

Following ACL reconstruction, graft inclination angles, ATT, and FA/TA were significantly greater than those of the contralateral knee joint (P<0.05). FTA and RTS did not differ significantly between sides, but exhibited significant correlation.

**Conclusions:**

Graft inclination angles could not fully recover to normal levels post-ACL reconstruction, while notable medial/lateral ATT occurred on the surgical side. Additionally, a significant correlation was observed between FTA and RTS, suggesting their potential as combined clinical indicators for assessing knee joint rotation stability.

## Introduction

Currently, the primary clinical treatment for anterior cruciate ligament (ACL) is the ACL reconstruction (ACLR), which has achieved satisfactory short-term and medium-term outcomes in patient satisfaction and knee stability. However, ACLR often does not fully restore the knee’s rotational stability [1]. In ACLR, successful surgery hinges on the precise positioning of the ACL graft bone tunnel, which determines the footprint of the femur and tibia, crucially influencing the graft’s inclination angles on both the sagittal and coronal planes.

Many current ACLR techniques aim to position the graft within the original ACL footprint for the purpose of restoring the normal knee joint stability [2]. However, in clinical practice, the variability of the arthroscopic technology has often led to the deviation of the graft footprint from the original ACL footprint [3]. The finite element method is used to simulate the scenarios when the femoral tunnel was positioned in the anterior and posterior directions and within ± 5mm at the far and near ends of the anatomical site, and the tibial tunnel was positioned in the anterior and posterior directions and within ± 5mm at the medial and lateral sides of the anatomical site. Reports indicate that a mere 5mm deviation in tunnel positioning can alter the ACL graft’s inclination angle significantly, ranging from 33.8° to 79.3° on the sagittal plane and from 56.5° to 85.6° on the coronal plane [3]. It is crucial to understand the impact of the changes in graft inclination angle caused by such surgical variation on the knee joint stability, because changes in knee joint stability can result in abnormal joint load, leading to the occurrence and progression of cartilage damage, and even the development of osteoarthritis [4]. Though ACL was reconstructed at the anatomical site, the inclination angle of the graft on the sagittal plane was variable when the knee joint was extended, which was related to the stability of the knee joint after surgery [5].

To identify more efficient quantitative indicators of knee stability, various exploratory studies have been conducted. Almekinders et al. [6] proposed the concept of anterior tibial translation (ATT) by observing the stress applied to the knee joint of ACLR patients through X-ray imaging, which emphasizes the relative anterior-posterior position relationship between the tibia and femur in a straight knee joint state. Tanaka et al. [7] found that the medial and lateral ATT of patients with ACL injury and patients with ACLR failure were significantly higher than those of the control subjects without ACL injury. Subsequently, more and more quantitative evaluations of the anterior-posterior position relationship of the femorotibial joint based on X-ray [8] or MRI [9] were reported, but the research on the rotational position relationship of the femorotibial joint remained scarce. Zhang et al. [10] used the difference between lateral ATT and medial ATT to represent the degree of tibial rotational subluxation (RTS), so as to evaluate the relative rotational relationship between the femur and tibia. Their results showed that the medial ATT, lateral ATT, and RTS in the ACL injury group were all higher than those in the healthy control group. Vassalou et al. [11] calculated the femorotibial angle (FTA) as the difference between the femoral rotation angle (FA) and tibial rotation angle (TA) to evaluate the rotational relationship of the tibia relative to the femur. The results showed that the FTA of patients with acute or chronic ACL injury was greater than that of healthy controls with intact ACL.

However, the existing literature lacks investigations on the comparison between the surgical and healthy sides of the same patient. Therefore, this study aimed to examine the differences in the graft inclination angle on the coronal plane and sagittal plane after ACLR relative to the healthy side of knee joint. Moreover, by measuring the medial and lateral ATT, FA and TA and calculating the FTA and RTS, the alignment relationship of the femorotibial joint was compared between the surgical and healthy sides.

## Materials and methods

**Fig 1:**
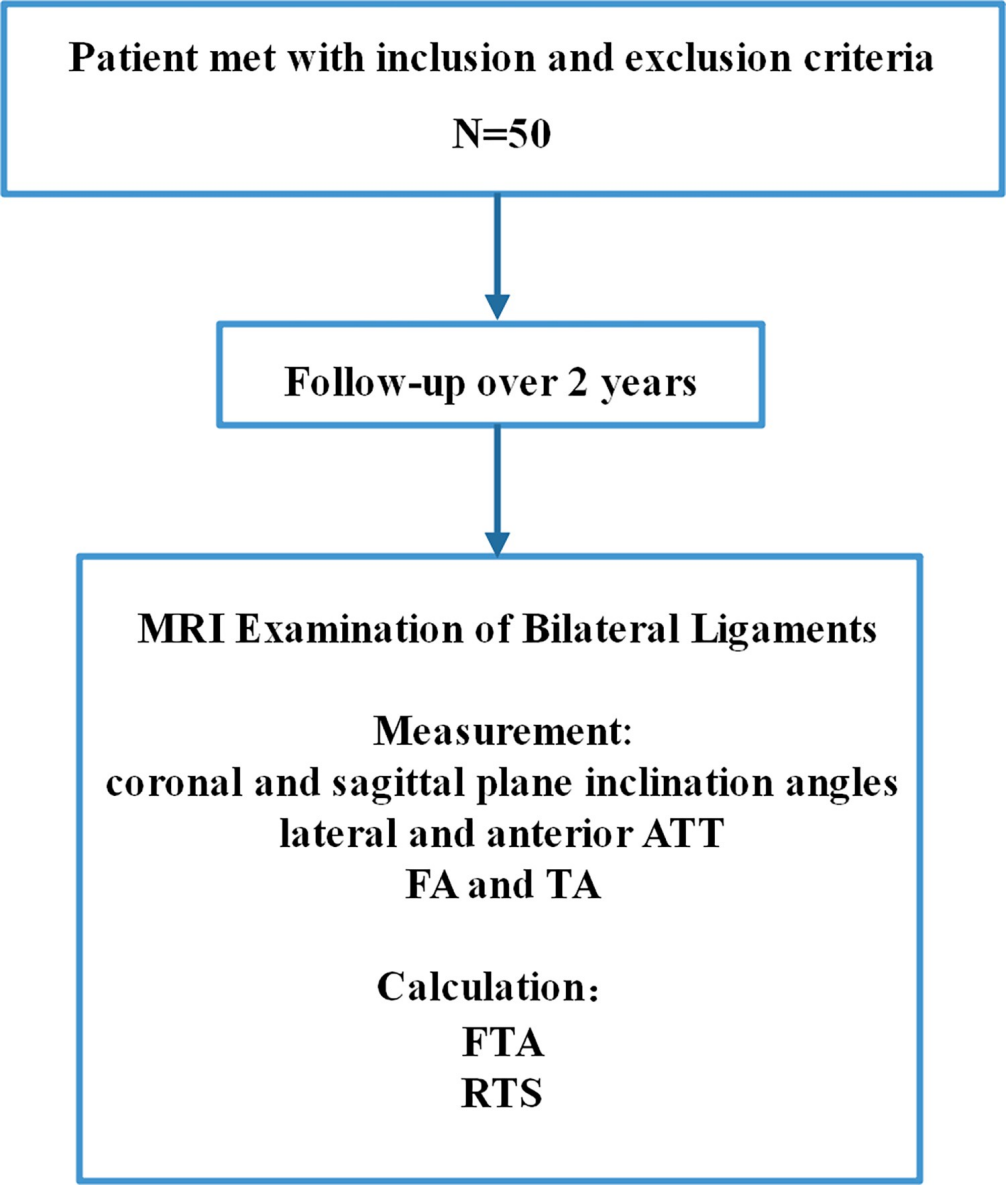
Flow diagram of the study design.

This retrospective study was approved by the ethics committee of Shenzhen Second People’s Hospital. Consent was obtained from each of the patients. The patients who underwent primary anatomical single-bundle ACLR in one surgery group (by Dr. W Zhu) at our department from June 2019 to June 2020 were reviewed. Patients would be included if they met the following criteria: (1) over 18 years of age; (2) primary ACL surgery; (3) no concomitant ligament injury; (4) unilateral ACL injury; (5) no previous surgery on the affected knee; (6) no chondral lesion worse than Outerbridge grade 2; and (7) ACL rupture confirmed clinically and by MRI. The exclusion criteria included: (1) damage of multiple ligaments or injury of articular cartilage; (2) radiographic evidence of Kellgren-Lawrence grade 3 or 4 osteoarthritis and/or severe osteoporosis; (3) ACL injuries of both sides of the knees; (4) partial ACL rupture; (5) concomitant total or subtotal meniscectomy; or (6) young patients with unclosed growth plates. Eventually, 50 patients were included in the present study. A flowchart of the patient selection process is presented in (Fig 1).

### Surgical technique

In accordance with our previous publication [12], we identified the lateral intercondylar and bifurcate ridges as crucial bony landmarks for femoral ACL attachment. The femoral tunnel, which should not surpass the lateral intercondylar ridge, was created in the center of the lateral bifurcate ridge. A K-wire was placed into the lateral femoral condyle at the 1:30 or 10:30 position through the AM portal using a freehand technique at 120° of knee flexion. With the K-wire as a guide, a femoral tunnel was reamed to the lateral cortex of the distal femur using a 4.5 mm EndoButton drill. A 30 mm femoral socket that matched the prepared graft diameter was then created using a cannulated reamer. The tibial tunnel was placed at the center of the ACL remnant through the AM surface of the tibia at the level of the tibial tubercle using a tibial guide (Smith & Nephew Acufex). The graft was first introduced into the tibial tunnel with a guide wire and then pulled directly into the femoral tunnel and fixed on the femoral side by flipping over the EndoButton (Smith & Nephew). The tibial side was fixed using a hydroxyapatite interference screw (DePuy Mitek) with a diameter 1 mm larger than the graft at 30° of knee flexion under 40 N of initial tension.

### MRI examinnation

Two years post-surgery, MRI examination of both surgical and contralateral sides were performed on these patients. They were instructed to refrain from physical activities the day prior to the MRI and to rest for 30 minutes beforehand, minimizing variance from different load conditions. The knee joint to be tested was placed within the coil and flexed 10°-15° for outward rotation fixation. MRI scans were performed by a 3.0T MRI instrument (Prisma, Siemens, Germany) using an orthogonal coil. We captured images of the sagittal plane, the coronal plane and the cross section. Scanning protocols encompassed T1WI spin echo and fat-suppressed T2WI sequences. The specific parameters were as follows: the oblique sagittal plane T1WI spin echo sequence (TR500ms, TE20ms); the oblique sagittal plane fat suppression T2WI sequence (TR3800ms, TE14ms); the coronal plane fat suppression T2WI sequence (TR4800ms, TE48ms); the cross-section fat suppression T2WI sequence (TR3200ms, TE77ms); layer thickness (3mm); layer spacing (0.3mm); FOV (160mm×160mm); matrix (320×256). After scanning, the data was transferred to the PACS workstation for measurement. Auxiliary lines were drawn on the corresponding cross-sectional view to measure the sagittal plane inclination angle, the coronal plane inclination angle, the medial ATT, and the lateral ATT. Then, the FA and TA were calculated accordingly. All imaging indicators were measured twice, with an interval of one month, and the average values were used for statistical analysis. Data collection spanned from July to December 2022.

### Measurement of coronal and sagittal plane inclination angles

**Fig 2:**
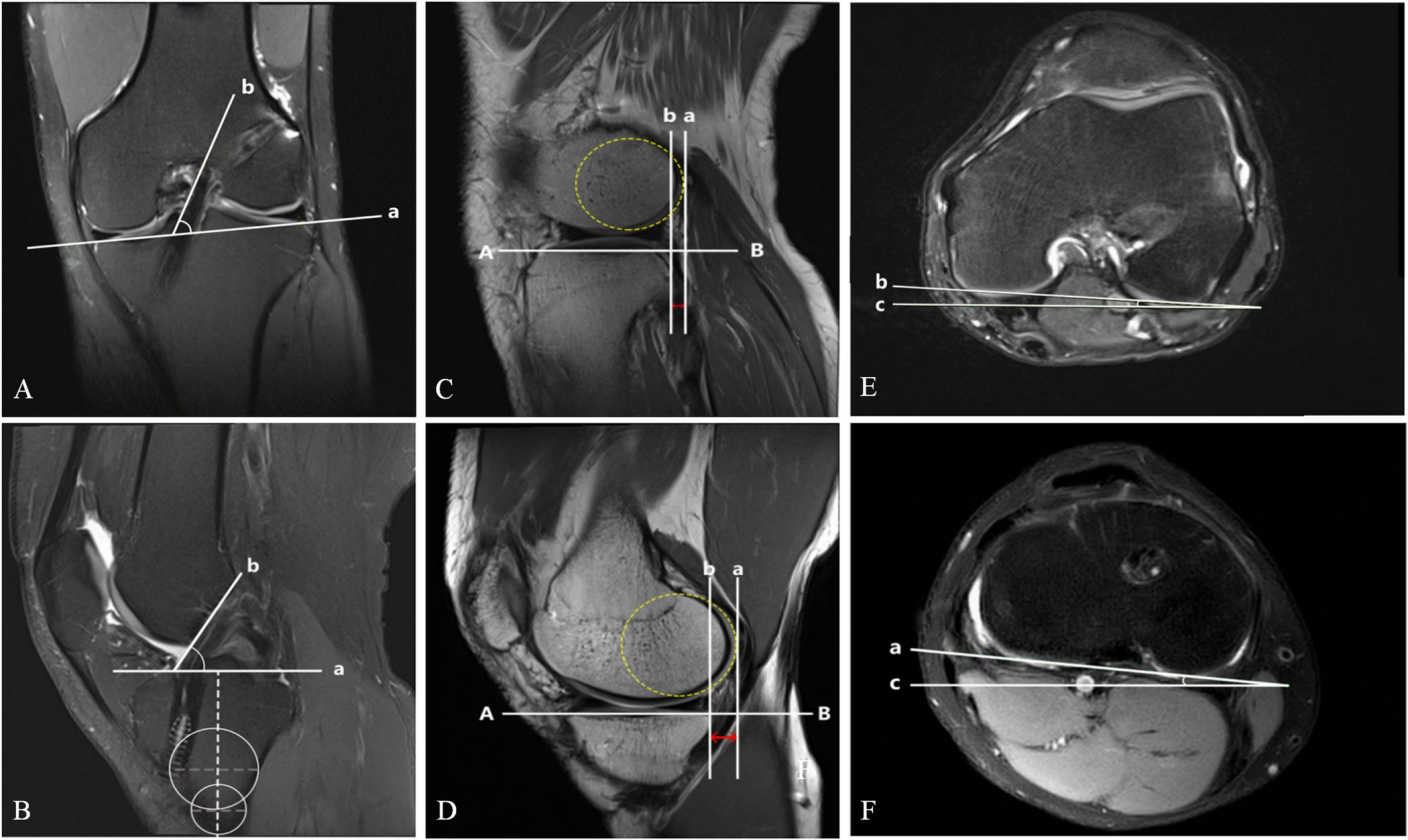
Measuring methods of various indicators based on MRI. Take the left knee joint as an example. (A) Coronal plane inclination angle; (B) Sagittal plane inclination angle; (C) Lateral ATT; (D) Medial ATT; (E) FA; (F) TA.

The coronal plane inclination angle refers to the included angle between the medial edge of ACL and a parallel line of the articular surface of tibial plateau. As the ACL cannot be fully visualized on a single coronal plane, tracing the medial edge requires multiple layers. First, the medial edge of the ACL was marked at the attachment point on the femoral side, and then the medial edge of the attachment point on the tibial side was marked. Subsequently, a line “b” was drawn along the medial edge of the ACL, connecting these marking points and forming an angle with line “a”, which is parallel to the articular surface of tibial plateau [13] (Fig 2A).

The sagittal plane inclination angle refers to the included angle between the anterior edge of ACL and a vertical line of the long axis of tibia. The layer showing the clearest ACL was used to mark line “b” formed by the anterior edge. The long axis of the tibia is defined by two circles. The diameter of the circle represents the width of the tibia. Then, a line connecting the centers of the two circles was drawn along the tibia shaft, with a perpendicular line “a” to line “b” [13]. The included angle formed by line “a” and “b” is the sagittal plane inclination angle (Fig 2B).

### Measurement of lateral and medial ATT

For the sagittal scanning, the layer just exposing the edge of fibular head was selected as the cross section for measurement. First, a line “AB” was formed by connecting the anterior and posterior edges of the lateral tibial plateau. Second, a line “b” that is perpendicular to “AB” and tangent to the posterior edge bone cortex of the lateral tibial plateau was drawn. Third, an optimal circle is drawn to conform to the contour of the lateral femoral condyle’s posterior edge. Fourth, a tangent line “a” of this most suitable circle that is perpendicular to “AB” was drawn. The vertical distance between line “a” and line “b” quantifies the lateral ATT [14]. The relative displacement of the tibia to the femur was recorded as positive, while the relative displacement of the femur to the tibia as negative (Fig 2C).

The central layer of the medial tibial plateau was chosen for cross-sectional measurement of medial ATT [14]. Similarly, the same method was used, selecting the anterior and posterior margins of the medial tibial plateau and the posterior margin of the medial femoral condyle as references (Fig 2D).

### Measurement of FA and TA

The layer with the maximum anteroposterior diameter of the femoral condyle was selected for measuring the FA. On the selected cross-sectional image, FA was measured by drawing a horizontal line “c” and a line “b” tangent to the posterior cortical edge of the femoral condyle [11]. A negative value of FA indicates inward rotation, while a positive value indicates outward rotation (Fig 2E).

The layer just above the fibular head was selected for measuring the TA. On the selected cross-sectional image, TA was measured by drawing a horizontal line “c” and a line “b” tangent to the posterior cortical edge of the tibial condyle [11]. A negative value of TA indicates inward rotation, while a positive value indicates outward rotation (Fig 2F).

### Calculation of FTA and RTS

FTA is defined as the difference between TA and FA [11], i.e., ∠aoc - ∠boc. A positive value of FTA indicates outward rotation of the tibia relative to the femur, while a negative value indicates inward rotation of the tibia relative to the femur. RTS is defined as the difference between lateral ATT and medial ATT [10].

### Statistical analysis

The present analysis was undertaken on a retrospective case series, classified as Level IV evidence. Study data were expressed as mean ± standard deviation (SD) and analyzed by SPSS 22.0 (IBM, USA). The imaging measurement parameters were tested by the paired samples t-test. Pearson correlation was employed to assess the relationship between RTS and FTA. All measurements were conducted using Adobe Photoshop CC 2018 (Adobe Inc., USA). The precision of measurement was determined according to the specific image quality. P<0.05 was considered to be statistically significant.

## Results

### Demographic characteristics

A total of 50 subjects were enrolled in this study, including 43 males and 7 females. The sample included 29 cases of left knee injury and 21 cases of right knee injury. The general demographic data were as follows: age, 30.42±6.45 years; height, 1.73±0.07cm; weight, 71.24±11.11kg; BMI, 23.33±4.65kg/m^2^. The study followed participants for over two years.

### Comparison of imaging indicators between the surgical and healthy sides

The inclination angle of the graft/ACL was measured by the MRI fat suppression T2WI sequence, and it was found that the average coronal plane inclination angle of the surgical side was greater than that of the healthy side (65.62±8.75° vs. 61.32±7.45°, P<0.05), and so was the average sagittal plane inclination angle (57.40±7.21° vs. 55.16±5.14°, P<0.05).

Sagittal MRI (T1WI sequence) revealed that the average lateral ATT was greater on the surgical side than on the contralateral side (5.29±3.54mm vs. 1.08±3.14mm, P<0.01). Meanwhile, the average anterior translation of the medial tibial plateau on the surgical side was also greater than that on the healthy side (3.42±3.26mm vs. -1.1±1.92mm, P<0.01). Calculating the difference between lateral ATT and medial ATT showed that the average RTS on the surgical side was lower than that on the healthy side (1.87±3.77mm vs. 2.18±3.57mm, P>0.05).

Based on measurements of the MRI fat suppression T2WI sequence, it was shown that the average FA on the surgical side was greater than that on the healthy side (0.30±5.49° vs. -1.78±2.76°, P<0.05). In addition, the average TA on the surgical side was smaller than that on the healthy side (-4.92±7.45° vs. -7.56±5.66°, P<0.05). The value of FTA was calculated as the difference between TA and FA, which indirectly reflects the rotation of the tibia relative to the femur. It was found that the average FTA on the surgical side was greater than that on the healthy side (-5.78±5.46° vs. -5.22±7.85°, P>0.05). Table 1 summarized the indicators of all MRI measurements.

**Table 1:** Comparison of imaging measurements in ACLR patients between the healthy and surgical sides.

Indicator	Healthy side (n = 50)	Surgical side (n = 50)	t	P
Coronal plane inclination angle (°)	61.32±7.45	65.62±8.75	-2.645	0.010*
Sagittal plane inclination angle (°)	55.16±5.14	57.4±7.21	-1.989	0.047*
Lateral ATT (mm)	1.08±3.14	5.29±3.54	-6.295	0.000**
Medial ATT (mm)	-1.1±1.92	3.42±3.26	-8.452	0.000**
RTS (mm)	2.18±3.57	1.87±3.77	0.422	0.674
FA (°)	-1.78±2.76	0.3±5.49	-2.393	0.019*
TA (°)	-7.56±5.66	-4.92±7.45	-1.956	0.049*
FTA (°)	-5.78±5.46	-5.22±7.85	-0.414	0.680

Note

**: P<0.001

*: P<0.05 indicate statistical significance.

The imaging measurement indicators were represented by *X±SD*. For FA and TA: a negative value indicates inward rotation, while a positive value indicates outward rotation. For FTA: a negative value indicates inward rotation of the tibia relative to the femur, while a positive value indicates outward rotation of the tibia relative to the femur.

**Fig 3:**
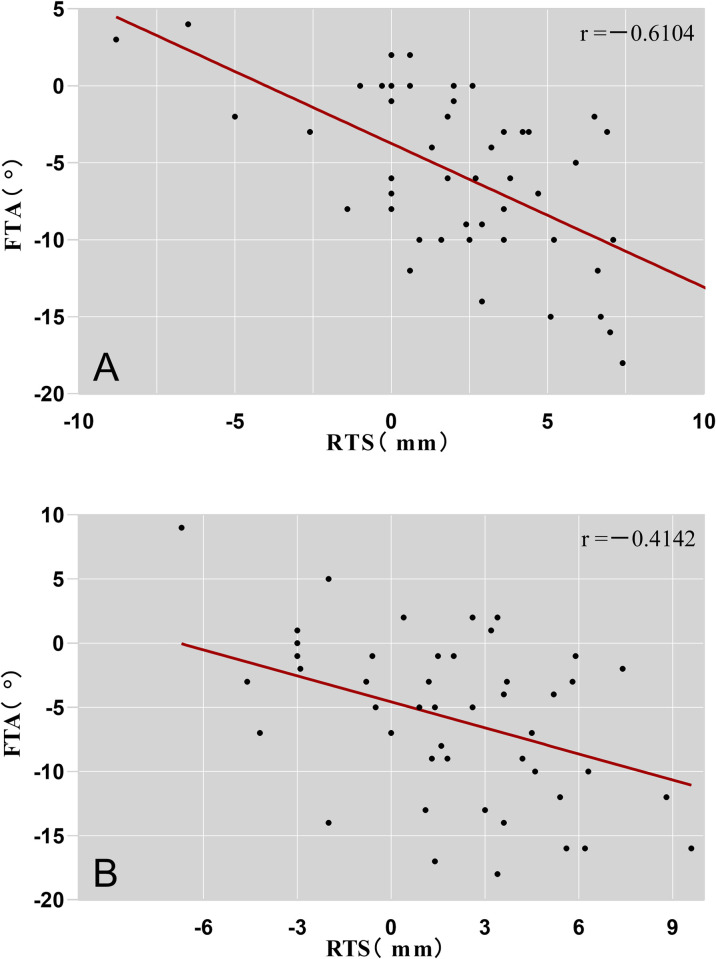
Correlation analysis between FTA and RTS on the healthy and surgical sides of the knee joint after ACLR. (A) Healthy side, (B) Surgical side.

Correlation analysis suggested that there was a significant negative correlation between RTS and FTA either on the healthy side (rp = -0.6104, P<0.001), or the surgical side of knee joint (r = -0.4142, P<0.001) (Fig 3).

## Discussion

In this study, it was found that the coronal and sagittal plane inclination angles of the knee joint on the operative side were larger than those on the healthy side, with both the medial and lateral tibial plateaus showing forward movement. At the same time, the good consistency of FTA and RTS makes it possible for us to evaluate the static rotation of the knee joint.

Previous studies have reported that the inclination angle of autologous ACL varies remarkedly. To be more specific, the inclination angle ranges from 43° to 59° on the sagittal plane and from 65° to 76° on the coronal plane [15–17]. The inclination angle of ACL was increasing throughout the entire growth and development stage; when the epiphysis was closed, the average inclination angle on the sagittal plane and coronal plane was 58.8° and 69.1°, respectively [18]. The results of our study showed that the average inclination angle on the sagittal plane in normal adults was 55.16°±5.14° (40°-67°), and that on the coronal plane was 61.32°±7.45° (47°-87°).

In the reconstructed ACL, the inclination angle of the graft is mainly determined by the starting points of the femoral and tibial tunnels and the course of the graft in the joint. The study found that changes in the anterior and posterior directions of the tibial tunnel had an impact on the course of the graft on the sagittal plane, while changes in the medial and lateral directions had an impact on the course of the graft on the coronal plane [18]. Besides, many other factors may also lead to changes in the inclination angle of the graft, including the choice of the graft [15], the choice of surgical technique [19]. A common finding of the aforementioned studies is that the sagittal plane inclination angle of the graft after surgical reconstruction is always greater than that of the ACL in the contralateral healthy knee joint. In our study, the average sagittal plane inclination angle of the graft was measured to be 57.4°, which was also greater than that of the ACL on the healthy side (55.16°), indicating that the knee joint after reconstruction can hardly recover to the state of the healthy knee joint. A series of failed ACLR cases and revealed that their graft orientation was more vertical than the native ACL. Therefore, the increase in the inclination angle of the graft after reconstruction may increase the risk of graft failure [20].

Moreover, we observed that the average coronal plane inclination angle of the graft was also greater than that of the ACL in the healthy knee joint (65.62° vs. 61.32°). Compared with the values in Fujimoto et al. [21] and Ahn et al. [15], the coronal plane inclination angle of the graft in our study was smaller. This is probably because the surgical method used in our study was the anteromedial approach technique, and independent femoral tunnel drilling at 120° knee flexion, positioning the bone tunnel within the native ACL’s femoral anatomical footprint, thereby better simulating the posterolateral bundle of the native ACL and better restoring the rotational stability of the knee joint. In contrast, the TT technique under arthroscope often cannot position the femoral tunnel within the anatomical footprint area [22]. More specifically, in the application of the TT technique, the tibial tunnel is positioned at the anatomical center of the tibial footprint, and the femoral tunnel is drilled through the tibial tunnel, resulting in the femoral tunnel being positioned slightly ahead of the original anatomical footprint area [23]. The positioning of these non-anatomical femoral tunnels tends to increase the graft’s inclination angle. Therefore, correctly positioning the femoral tunnel within the ACL’s anatomical footprint requires a larger drilling angle relative to the medial wall of the lateral femoral condyle.

Therefore, an increased PTS can lead to anterior displacement of the tibia relative to the femur [24]. In our study, all patients exhibited a PTS of less than 15°, with no detected damage to the posterior roots of the medial or lateral meniscus. Thus, the potential impact from the above factors was avoided. Even after undergoing ACLR, ATT often cannot be restored, leading to changes in the alignment of the femorotibial joint [25] The above research findings have drawn wide concern from scholars regarding the effect of ACLR, because changes in the position of the tibia would have a negative impact on the recovery of normal kinematics of the knee joint and the improvement of postoperative stability [26–28].

In this study, the measurements of medial and lateral ATT indicated that both indicators were increased in patients after ACLR compared with the healthy control group. Snoj et al. [14] reported the same results in their study, but the specific medial and lateral ATT values they observed were greater than those in ours. Nonetheless, the common findings of the two suggest that the tibia moves forward relative to the femur after ACLR. Although ACLR can reduce the sagittal plane relaxation to the normal range, it cannot restore the normal femorotibial alignment [29]. Persistent anterior tibial displacement under static conditions may partially explain the not-reduced incidence of osteoarthritis post-ACLR [30].

To date, there are few studies that evaluated the femorotibial rotational relationship after ACLR based on MRI. However, we observed that the postoperative FTA values of the surgical side were basically consistent with those of the healthy side (-5.22±7.85° vs. -5.78±5.46°), suggesting that the relative femorotibial rotational relationship had returned to normal after ACLR. Although our results showed significant differences in FA and TA between the surgical and healthy sides after ACLR, evaluating the femorotibial position should not rely solely on a single indicator, because a single indicator can be greatly affected by the knee joint position during examination. In this regard, FTA can indirectly reflect the position relationship of the tibia relative to the femur by utilizing a horizontal line in the MRI section as the reference, and has become an effective indicator for evaluating the static rotational stability of the knee joint in imaging examination.

In addition, in vivo and in vitro studies have revealed a correlation between ATT and the tibial inward rotation relative to the femur [31]. Fukubayashi et al. [32] showed in vitro that ACL rupture would double the ATT, but the phenomenon of tibial inward rotation relative to the femur disappeared accordingly. The above results suggest that ACL is closely related to the control of anteroposterior and rotational stability. In this study, we described the anteroposterior position relationship between the tibia and femur after ACLR by measuring the lateral and medial ATT on the sagittal plane based on MRI images. More importantly, we also quantified the rotational relationship between the tibia and femur using the indicator of RTS by calculating the difference between the lateral and medial ATT.

During correlation analysis, the existence of such a negative correlation between FTA and RTS reflects the consistency between these two indicators in evaluating the femorotibial rotational relationship. The simultaneous application of FTA and RTS in clinical examination can significantly enhance the credibility of the evaluation results in assessing the femorotibial relationship of the knee joint. In future research, we will further verify the consistency between static femorotibial rotational relationships on MRI and dynamic knee joint rotation, enhancing the evaluation of postoperative rotational stability via MRI.

Our study has been disseminated as a preprint at the following link [33].

## Limitations

Our study, while providing valuable insights, has certain limitations due to its retrospective design. This design constraint limits our ability to perform detailed assessments of anteroposterior instability, a factor important for accurately evaluating knee rotational stability. Consequently, this could influence the interpretability of our findings. Moreover, the retrospective approach may not fully capture the diversity of the general population undergoing ACLR, potentially affecting the generalizability of our results. Additionally, despite adhering to a standardized MRI protocol, minor variations in patient posture or knee flexion during the examination could introduce inconsistencies. Future studies, possibly with prospective designs, are recommended to overcome these limitations and confirm our findings, enhancing the reliability and applicability of the results.

## Conclusions

After ACLR, the inclination angle of the graft cannot be restored to the level of the healthy knee joint, and notable medial and lateral ATT occurred on the surgical side compared to the healthy side of the knee joint. In addition, there is a significant correlation between FTA and RTS, which are expected to be used as combined clinical indicators for evaluating the stability of knee joint rotation.
